# Repetitive navigated transcranial magnetic stimulation to facilitate motor rehabilitation in acute pediatric hemorrhagic stroke – illustrative case

**DOI:** 10.1016/j.bas.2025.104397

**Published:** 2025-09-08

**Authors:** Maximilian Schwendner, Haosu Zhang, Leonie Kram, Mohammed Issa, Ahmed El Damaty, Sandro M. Krieg, Sebastian Ille

**Affiliations:** Department of Neurosurgery, Heidelberg University Hospital, Ruprecht-Karls-University, Heidelberg, Germany

**Keywords:** Arteriovenous malformation, Repetitive transcranial magnetic stimulation, Neuromodulation, Pediatric stroke

## Abstract

**Background:**

Hemorrhage from arteriovenous malformations (AVMs) represents the most frequent cause of pediatric hemorrhagic stroke. However, outcomes often bear permanent extensive neurological impairments.

**Observations:**

An eleven-year-old male presented to the emergency department following a sudden onset of left-sided hemiparesis and continued loss of consciousness after an epileptic seizure. Imaging revealed a large hemorrhagic stroke of the right frontal lobe resulting from bleeding of an arteriovenous malformation. After microsurgical resection, low-frequency repetitive navigated transcranial magnetic stimulation (nrTMS) therapy targeting the motor hotspot of the unaffected hemisphere was administered. At the 4-month follow-up visit, the patient recovered motor function, with persisting minor deficits in leg mobility and a predominantly distant arm paresis.

**Lessons:**

Utilizing nrTMS in pediatric patients effectively assessed motor function and in addition potentially contributed to early-stage motor rehabilitation. This method marks a potential advancement in acute pediatric stroke management.

## Highlights

• Key considerations for the use of navigated transcranial magnetic stimulation (nTMS) in pediatric patients are discussed.
• The application of nrTMS for motor rehabilitation in an 11-year-old patient with acute hemorrhagic stroke is presented.
• The therapeutic principles of nrTMS for pediatric patients are illustrated.
• The potential of nrTMS as a motor rehabilitation therapy in pediatric patients is explored.

## Abbreviations:

AVM: arteriovenous malformation
CT: computed tomography
ICP: intracranial pressure
MEPs: motor-evoked potentials
MRI: magnetic resonance imaging
nTMS: navigated transcranial magnetic stimulation
nrTMS: repetitive navigated transcranial magnetic stimulation
rTMS: repetitive transcranial magnetic stimulation
TMS: transcranial magnetic stimulation

## 1 Introduction

Arteriovenous malformations (AVMs) are predominantly congenital but often manifest during adulthood (Di Rocco et al., 2000). In pediatric cases, brain AVMs commonly manifest through hemorrhagic strokes, constituting the leading cause of pediatric hemorrhagic stroke and resulting in poor outcomes with high rates of morbidity and mortality (Di Rocco et al., 2000; Smith et al., 2002; Antkowiak et al., 2021). Surgical excision is typically the preferred treatment for parenchymal AVMs in children (Di Rocco et al., 2000; Antkowiak et al., 2021).

In general, in stroke patients, the brain, and, in particular, white matter pathways, such as the corticospinal tract (CST), are damaged by ischemia. Still, there is a potential to recover from the concluding functional disability. Ipsilesional mechanisms have been identified that support motor recovery (Fridman et al., 2004; Werhahn et al., 2003). Moreover, as shown in studies of stroke patients, the motor cortex of the contralesional hemisphere, especially, contributes to ipsilesional recovery through interhemispheric interactions (Carter et al., 2010; Floel et al., 2008). A central mechanism that impairs motor recovery is the enhanced transcallosal inhibition (TCI) of the affected hemisphere by the unaffected hemisphere (Duque et al., 2005; Murase et al., 2004). Thus, the non-invasive downregulation of the contralesional motor cortex enables ipsilesional motor recovery by reducing TCI (Hummel and Cohen, 2006).

Transcranial magnetic stimulation (TMS) is a non-invasive and reliable tool for the modulation of brain activity (Lefaucheur et al., 2014; Siebner and Rothwell, 2003). Repetitive TMS (rTMS) can up- or down-regulate cortical excitability; low-frequency rTMS can down-regulate the ipsilateral motor cortex and enhance the cortical excitability of the contralateral hemisphere (Maeda et al., 2000; Kobayashi et al., 2004). In adult stroke patients, low-frequency rTMS of the contralesional motor cortex has been applied successfully and has proven beneficial (Graef et al., 2016; Hao et al., 2013; Hsu et al., 2012; Le et al., 2014).

Meanwhile, repetitive navigated transcranial magnetic stimulation (nrTMS) has also been successfully applied for motor rehabilitation in an acute setting in adult glioma patients suffering postoperative paresis following ischemic events during surgery (Ille et al., 2021).

While nrTMS has proven effective in treating acute postoperative ischemic neurological deficits in adults, its application in children remains largely unexplored. Limited evidence is available, primarily focusing on chronic conditions resulting from ischemic lesions such as those seen in cerebral palsy (Ille et al., 2021; Kirton et al., 2016; He et al., 2024; Marzbani et al., 2018). This case describes the application of nrTMS in a child suffering from acute hemorrhage stroke due to bleeding from a brain AVM.

## 2 Material and methods

### 2.1 Patient informed consent

The research adhered to the principles outlined in the Declaration of Helsinki. The decision to pursue an individual treatment attempt was made in consultation with the child's parents. Informed consent was obtained from the parents by institutional guidelines and ethical standards.

### 2.2 Inclusion criteria and safety consideration

The patient showed acute extensive neurological deficit related to ischemic injury. The patient had no contraindications for nTMS or magnetic resonance imaging. Therapy was performed according to safety guidelines for nTMS in children (Rossi et al., 2009, 2021).

### 2.3 Navigated transcranial magnetic stimulation

The rationale for nTMS over TMS was, that nTMS allows for accurate motor mapping as well as highly accurate repetitive stimulation, minimizing potential discomfort and optimizing accuracy, especially in children with reduced tolerability, postoperative and variable head sitze in children. An nTMS motor mapping examination was conducted to further evaluate the motor system's integrity. A contrast-enhanced T1-weighted MRI sequence was imported to a Nexstim eXimia NBS system, version 5.2 (Nexstim Plc., Helsinki, Finland). Motor mapping of both hemispheres was performed before therapy and during follow-up according to clinical routine (Krieg et al., 2017). For the cortical representations of the upper extremity, an intensity of 110 % of the individual resting motor threshold (rMT) was applied. In contrast, using single-pulse application, at least 130 % of the rMT was used to stimulate the lower extremity (Krieg et al., 2017).

**Fig. 1:**
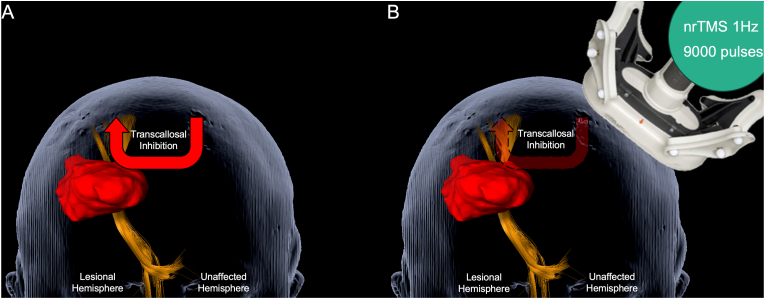
Treatment principle. Fig. 1 illustrates the principle of the transcallosal inhibition of the unaffected hemisphere (A) and the treatment strategy of repetitive navigated transcranial magnetic stimulation (nrTMS) to inhibit the dysregulated transcallosal inhibition and facilitate motor recovery (B).

To facilitate optimal motor recovery, a repetitive nTMS protocol was performed on the non-lesional hemisphere's motor hotspot (Ille et al., 2021) (Fig. 1). A low-frequency nrTMS stimulation of 1 Hz was performed for 15 min (900 pulses) at an intensity of 110 % of the resting motor threshold (Ille et al., 2021). nTMS treatment was performed according to clinical routine by an experienced physician, the patient was constantly monitored during therapy and free of seizures during the course of therapy. Therapy was conducted for eight consecutive days, each followed by physiotherapy.

## 3 Results

**Fig. 2:**
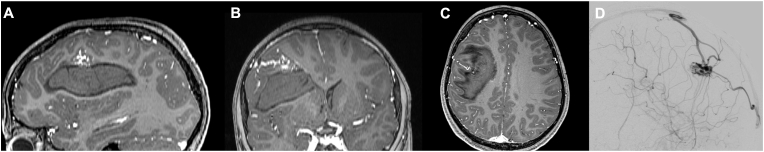
Preoperative Imaging. The preoperative MRI scan showing the extensive intracerebral hemorrhage of the right frontal lobe is illustrated in sagittal (A), coronal (B), and axial (C) views. In addition, a preoperative angiography (D) was performed.

An 11-year-old boy without any known previous medical history presented himself via the emergency department following a sudden onset of a left-sided hemiparesis and continued loss of consciousness after an epileptic seizure with a Glasgow Coma Scale score of 7. CT- and MRI-imaging revealed a large intracerebral hemorrhage within the right frontal lobe attributed to an underlying arteriovenous malformation (Fig. 2). Subsequently, an angiogram revealed an AVM classified as Spetzler Martin °2 with a main feeder of the superior truncus of the middle cerebral artery and venous drainage into the superior sagittal sinus (Fig. 2) (Spetzler and Martin, 1986).

An intracranial pressure (ICP) probe was placed, which showed elevated ICP. The patient underwent emergency surgery to evacuate the mass occupying hemorrhage and to resect the AVM. Postoperative angiograms and MRI indicated a complete resection of the AVM, while postoperative MRI revealed perilesional subcortical ischemia. Postoperatively, the patient was closely monitored at our children's intensive care unit. In the neurological exam after extubation, the patient exhibited left-sided hemiplegia, facial palsy, anesthesia of the arm, and dysphagia, with a PedNIHSS of 19. The patient was transferred to the intermediate care unit on the sixth postoperative day for further management with a PedNIHSS of 13.

**Fig. 3:**
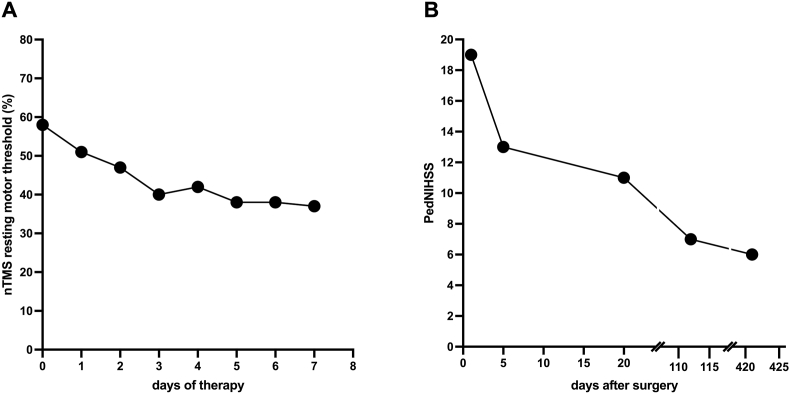
Parameters during therapy Fig. 3 shows a timeline the resting motor threshold of the unaffected hemisphere of all consecutive days during repetitive navigated transcranial magnetic stimulation (A). Furthermore, the Pediatric National Institutes of Health Stroke Scale (PedNIHSS) scores are arranged on a timeline for the day of hemorrhage, 5th day after hemorrhage, 20th day after hemorrhage as well as the status at the follow-up examinations after four and fourteen months (B).

We performed navigated transcranial magnetic stimulation (nTMS) motor mapping to further evaluate the integrity of the motor system. No motor-evoked potentials (MEPs) could be elicited from the upper or lower extremities while mapping the affected and the contralateral hemisphere. A nrTMS protocol was performed on the non-lesional hemisphere's motor hotspot to facilitate optimal motor recovery. The resting motor threshold of the non-lesional hemisphere, as an indirect measure of cortical excitability of the primary motor area, gradually decreased from 58 % before the initial nrTMS session to 37 % by the final session (Fig. 3). The stimulation sessions were well received, and no adverse events were observed. At the follow-up nTMS motor mapping performed at the last session of nrTMS, no MEPs were found.

On the 20th postoperative day, the patient was discharged to a neurological rehabilitation unit with a PedNIHSS of 11. Up to that point, the patient showed a partial recovery of the motor function of the leg, while no motor function of the arm was observed (Fig. 3).

**Fig. 4:**
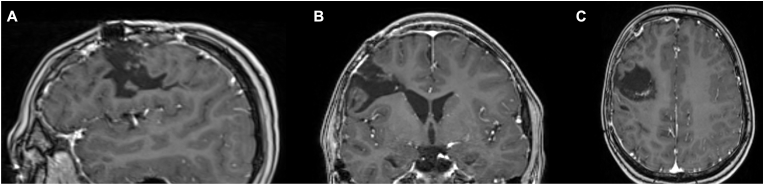
Follow-up MRI. The follow-up MRI showing no remnants of the arteriovenous malformation is depicted in sagittal (A), coronal (B), and axial (C) views.

**Fig. 5:**
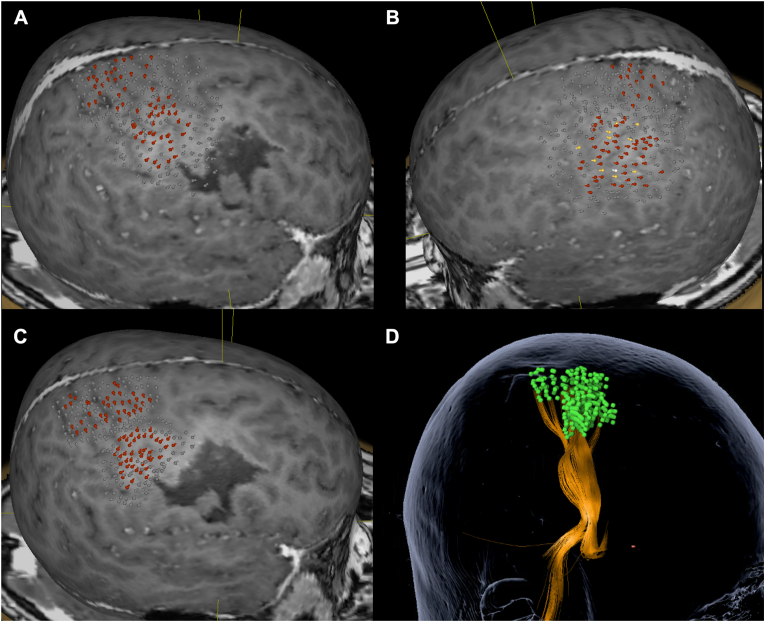
nTMS motor mapping indicating motor recovery at follow-up. Fig. 5 illustrates the navigated transcranial magnetic stimulation (nTMS) motor maps of the lesional (A) and unaffected (B) hemispheres at the follow-up examination four months after hemorrhage. The presence of nTMS-positive stimulation sites for the upper and lower extremities (illustrated depending on the corresponding voltage of the elicited motor-evoked potentials: red = 50–500 μV; yellow = 500–1000 μV; white = ≥1 mV; gray = ≤50 μV or no response) corresponded with the patient's recovery in motor function. Motor mapping of the lesional hemisphere was repeated at follow-up fourteen months after the hemorrhage (C), including function-based fibertracking of the corticospinal tract (D).

At the follow-up examination conducted four months post-hemorrhage, the patient demonstrated a partial recovery of motor function, exhibiting the regained capability to walk and execute voluntary movements with the left arm and a partial recovery in sensory function (Fig. 4, Fig. 5). Nevertheless, a significant paresis of the left hand persisted at follow-up four and fourteen months after the bleeding, with a PedNIHSS of 7 and 6 (Fig. 3).

Corresponding with the clinical recovery of motor function, we objectified motor rehabilitation by the means of nTMS motor mapping. While no MEPs on the lesional hemisphere were elicited during nrTMS therapy, we were able to elicit MEPs of proximal and distal muscle representations of the upper extremities and muscle representations of the lower extremities during follow-up (Fig. 5).

## 4 Discussion

This report illustrates the application of nrTMS for the therapy of motor deficits in an eleven-year-old suffering from an acute ischemic lesion resulting from bleeding and surgical treatment of a brain AVM in children.

In this case, nTMS was chosen as a diagnostic and therapeutic tool. Other diagnostic tools, such as functional MRI to assess the motor system, were not available with reasonable effort, as the patient required extensive sedation during MRI scanning. Therefore, imaging was limited to minimal clinically needed diagnostics.

Low-frequency rTMS has already been successfully applied in the treatment of children with attention deficit and hyperactivity disorder aged seven to twelve years (Gomez et al., 2014). Regarding treating motor impairments, positive effects of rTMS in children have been observed for low-frequency rTMS of 1 Hz to the unaffected hemisphere for treating cerebral palsy (He et al., 2024; Marzbani et al., 2018). A blinded, randomized-controlled trial by Kirton et al. assessed low-frequency rTMS of 1Hz applied to the contralateral motor hotspots in children aged 6–19 years suffering from motor deficits due to unilateral perinatal ischemic stroke (Kirton et al., 2016). The outcomes revealed improvements in motor function, affirming rTMS as a viable and safe therapeutic approach (Kirton et al., 2016). Current theories in both pediatric and adult stroke suggest that an imbalance exists between activation of the lesional and non-lesional hemispheres. Accordingly, potential targets for neuromodulation include stimulation of the lesional or perilesional motor cortex, or inhibition of the non-lesional motor cortex, with the aim of enhancing or restoring cortical motor control in favor of the lesioned hemisphere (Kirton, 2013; Pascual et al.bib_pascualleone_et_al_2005, 2005).

In the presented case, neurological deficits were primarily caused by the hemorrhagic lesion and subsequent local mass effect and not by the surgical intervention itself. However, an acute onset of ischemia-related deficits was present as well.

Based on the above-mentioned previous reports in the literature on low-frequency rTMS and our own clinical experience, we applied our established protocol of nrTMS in this particular case, which was well tolerated. Contrary to previous studies, rTMS was conducted with the addition of electric-field navigation to ensure optimal targeting of the motor hotspot and applied in a patient suffering from an acute ischemic lesion by a hemorrhagic stroke.

Several specific factors necessitate consideration when considering the application of transcranial magnetic stimulation TMS in pediatric populations. These include the maturation of cortical excitability, closure of the fontanelle, and the growth of the external auditory canal (Di Rocco et al., 2000). Expert guidelines conclude that single-pulse and paired-pulse TMS are safe for children two years and older (Rossi et al., 2009, 2021). Regarding repetitive rTMS, there is currently a lack of data on the potential adverse effects. This leads to the conclusion that rTMS in children should only be used to treat neurological disorders (Rossi et al., 2009, 2021).

An inherent property of the developing brain in pediatrics is increased plasticity (Anderson et al., 2011; Johnston, 2009). The developing brain is suggested to show better recovery capacity after early brain insults such as stroke due to rapid synaptogenesis, increased myelination, and faster reorganization processes of neuronal networks during this period (Anderson et al., 2011; Johnston, 2009). On the contrary, the developing brain is particularly vulnerable to early brain insult, which may lead to unfavorable outcomes due to disrupted brain development (Anderson et al., 2011; Johnston, 2009). Therefore, neither plasticity nor vulnerability theories can thoroughly explain the range of functional outcomes after an early brain insult (Anderson et al., 2011)^,^(deVeber et al., 2000).

However, the isolated effect of nrTMS on motor recovery in the presented single-case report cannot be determined, since there are no established electrophysiological parameters to assess the impact of nrTMS, especially in children, and can therefore only be considered as a feasibility report in one case. Since this is a single case, the reported findings might be subject to placebo effects, or the natural course of the disease including juvenile cortical plasticity, and therefore, results may not be generalized. In general, most children show a higher potential for functional recovery after severe neurological deficits compared to adults with the aid of physiotherapy, which could be affecting our outcome (Kim et al., 2009). Further, more extensive studies and possibly randomized controlled trials with standardized nrTMS protocols, outcome measures such as rMT, longitudinal motor mapping, and detailed clinical assessment are required to prove the benefit or as a reliable tool for neurorehabilitation in pediatric patients, since the potential of nrTMS remains underexplored, especially for this special patient group.

## 5 Conclusions

This case represents the first application of nrTMS for the treatment of acute hemiparesis in pediatric patients following a hemorrhagic stroke secondary to a ruptured brain AVM. Utilizing nTMS, a non-invasive technique, proved effective in assessing motor function on the one hand and potentially contributed to facilitating early-stage motor rehabilitation on the other hand. This method marks a potential advancement in acute pediatric stroke management.

## Author contributions

Conceptualization: AED, SMK, SI; Methodology: SI, SMK, MS; Formal analysis and investigation: MS, HZ, LK, MI; Writing - original draft preparation: MS; Writing - review and editing: MS, HZ, LK, MI, AED, SMK, SI; Resources: SMK, SI; Supervision: SI.

All authors agreed on the publication of the final version of this manuscript.

## Data availability statement

The authors declare that the data supporting the findings of this study are available within the paper and its materials.

## Declaration of competing interest

The authors declare the following financial interests/personal relationships which may be considered as potential competing interests: Maximilian Schwendner reports a relationship with Level Ex Incorporated that includes: consulting or advisory. Maximilian Schwendner reports a relationship with Sonovum GmbH that includes: travel reimbursement. Sebastian Ille reports a relationship with Brainlab AG that includes: consulting or advisory. Sebastian Ille reports a relationship with Nexstim Oy that includes: consulting or advisory. Sebastian Ille reports a relationship with Carl Zeiss Meditec AG that includes: consulting or advisory. Sandro M. Krieg reports a relationship with Brainlab AG that includes: consulting or advisory. Sandro M. Krieg reports a relationship with Ulrich Medical GmbH that includes: consulting or advisory. Sandro M. Krieg reports a relationship with Need Inc that includes: consulting or advisory and equity or stocks. If there are other authors, they declare that they have no known competing financial interests or personal relationships that could have appeared to influence the work reported in this paper.
