# Supplementary material for: Repetitive navigated transcranial magnetic stimulation to facilitate motor rehabilitation in acute pediatric hemorrhagic stroke – illustrative case
Source: Brain Spine. 2025 Sep 8;5:104397. doi: 10.1016/j.bas.2025.104397 (PMC12455120; doi:10.1016/j.bas.2025.104397)
Supplement: Multimedia component 1 [file mmc1.docx]

**Repetitive navigated transcranial magnetic stimulation to facilitate motor rehabilitation in acute pediatric hemorrhagic stroke – illustrative case**

Maximilian Schwendner, MD; Haosu Zhang, MD, PhD; Leonie Kram, MSc; Mohammed Issa, MD; Ahmed El Damaty, MD; Sandro M. Krieg MD, MBA; Sebastian Ille, MD

Department of Neurosurgery, Heidelberg University Hospital, Ruprecht-Karls-University; Heidelberg, Germany

Discussion

This report illustrates the application of nrTMS for the therapy of motor deficits in an eleven-year-old suffering from an acute ischemic lesion resulting from bleeding and surgical treatment of a brain AVM in children.

In this case, nTMS was chosen as a diagnostic and therapeutic tool. Other diagnostic tools, such as functional MRI to assess the motor system, were not available with reasonable effort, as the patient required extensive sedation during MRI scanning. Therefore, imaging was limited to minimal clinically needed diagnostics.

Low-frequency rTMS has already been successfully applied in the treatment of children with attention deficit and hyperactivity disorder aged seven to twelve years^23^. Regarding treating motor impairments, positive effects of rTMS in children have been observed for low-frequency rTMS of 1 Hz to the unaffected hemisphere for treating cerebral palsy^24,25^. A blinded, randomized-controlled trial by Kirton et al. assessed low-frequency rTMS of 1Hz applied to the contralateral motor hotspots in children aged 6 to 19 years suffering from motor deficits due to unilateral perinatal ischemic stroke^20^. The outcomes revealed improvements in motor function, affirming rTMS as a viable and safe therapeutic approach^20^.

In the presented case, neurological deficits were primarily caused by the hemorrhagic lesion and subsequent local mass effect and not by the surgical intervention itself. However, an acute onset of ischemia-related deficits was present as well.

Based on the above-mentioned previous reports in the literature on low-frequency rTMS and our own clinical experience, we applied our established protocol of nrTMS in this particular case, which was well tolerated. Contrary to previous studies, rTMS was conducted with the addition of electric-field navigation to ensure optimal targeting of the motor hotspot and applied in a patient suffering from an acute ischemic lesion by a hemorrhagic stroke.

Several specific factors necessitate consideration when considering the application of transcranial magnetic stimulation TMS in pediatric populations. These include the maturation of cortical excitability, closure of the fontanelle, and the growth of the external auditory canal ^1^. Expert guidelines conclude that single-pulse and paired-pulse TMS are safe for children two years and older^26,27^. Regarding repetitive rTMS, there is currently a lack of data on the potential adverse effects. This leads to the conclusion that rTMS in children should only be used to treat neurological disorders^26,27^.

An inherent property of the developing brain in pediatrics is increased plasticity^28,29^. The developing brain is suggested to show better recovery capacity after early brain insults such as stroke due to rapid synaptogenesis, increased myelination, and faster reorganization processes of neuronal networks during this period^28,29^. On the contrary, the developing brain is particularly vulnerable to early brain insult, which may lead to unfavorable outcomes due to disrupted brain development^28,29^. Therefore, neither plasticity nor vulnerability theories can thoroughly explain the range of functional outcomes after an early brain insult ^28,30^.

However, the isolated effect of nrTMS on motor recovery in the presented case cannot be determined since there are no established electrophysiological parameters to assess the impact of nrTMS, especially in children. Based on previous literature, a supportive effect of nrTMS on neurological recovery can be assumed. In general, most children show a higher potential for functional recovery after severe neurological deficits compared to adults with the aid of physiotherapy, which could be affecting our outcome^31^. Further, more extensive studies and possibly randomized controlled trials are required to prove the benefit or as a reliable tool for neurorehabilitation in pediatric patients.

References

1. Di Rocco C, Tamburrini G, Rollo M. Cerebral arteriovenous malformations in children. *Acta Neurochir (Wien)*. 2000;142(2):145-56; discussion 156-8. doi:10.1007/s007010050017

2. Smith ER, Butler WE, Ogilvy CS. Surgical approaches to vascular anomalies of the child's brain. *Curr Opin Neurol*. Apr 2002;15(2):165-71. doi:10.1097/00019052-200204000-00007

3. Antkowiak L, Putz M, Rogalska M, Mandera M. Multimodal Treatment of Pediatric Ruptured Brain Arteriovenous Malformations: A Single-Center Study. *Children (Basel)*. Mar 11 2021;8(3)doi:10.3390/children8030215

4. Fridman EA, Hanakawa T, Chung M, Hummel F, Leiguarda RC, Cohen LG. Reorganization of the human ipsilesional premotor cortex after stroke. *Brain : a journal of neurology*. Apr 2004;127(Pt 4):747-58. doi:10.1093/brain/awh082

5. Werhahn KJ, Conforto AB, Kadom N, Hallett M, Cohen LG. Contribution of the ipsilateral motor cortex to recovery after chronic stroke. *Annals of neurology*. Oct 2003;54(4):464-72. doi:10.1002/ana.10686

6. Carter AR, Astafiev SV, Lang CE, et al. Resting interhemispheric functional magnetic resonance imaging connectivity predicts performance after stroke. *Annals of neurology*. Mar 2010;67(3):365-75. doi:10.1002/ana.21905

7. Floel A, Hummel F, Duque J, Knecht S, Cohen LG. Influence of somatosensory input on interhemispheric interactions in patients with chronic stroke. *Neurorehabilitation and neural repair*. Sep-Oct 2008;22(5):477-85. doi:10.1177/1545968308316388

8. Duque J, Hummel F, Celnik P, Murase N, Mazzocchio R, Cohen LG. Transcallosal inhibition in chronic subcortical stroke. *Neuroimage*. Dec 2005;28(4):940-6. doi:10.1016/j.neuroimage.2005.06.033

9. Murase N, Duque J, Mazzocchio R, Cohen LG. Influence of interhemispheric interactions on motor function in chronic stroke. *Ann Neurol*. Mar 2004;55(3):400-9. doi:10.1002/ana.10848

10. Hummel FC, Cohen LG. Non-invasive brain stimulation: a new strategy to improve neurorehabilitation after stroke? *The Lancet Neurology*. Aug 2006;5(8):708-12. doi:10.1016/s1474-4422(06)70525-7

11. Lefaucheur JP, Andre-Obadia N, Antal A, et al. Evidence-based guidelines on the therapeutic use of repetitive transcranial magnetic stimulation (rTMS). *Clinical neurophysiology : official journal of the International Federation of Clinical Neurophysiology*. Nov 2014;125(11):2150-2206. doi:10.1016/j.clinph.2014.05.021

12. Siebner HR, Rothwell J. Transcranial magnetic stimulation: new insights into representational cortical plasticity. *Experimental brain research*. Jan 2003;148(1):1-16. doi:10.1007/s00221-002-1234-2

13. Maeda F, Keenan JP, Tormos JM, Topka H, Pascual-Leone A. Modulation of corticospinal excitability by repetitive transcranial magnetic stimulation. *Clinical neurophysiology : official journal of the International Federation of Clinical Neurophysiology*. May 2000;111(5):800-5.

14. Kobayashi M, Hutchinson S, Theoret H, Schlaug G, Pascual-Leone A. Repetitive TMS of the motor cortex improves ipsilateral sequential simple finger movements. *Neurology*. Jan 13 2004;62(1):91-8. doi:10.1212/wnl.62.1.91

15. Graef P, Dadalt MLR, Rodrigues D, Stein C, Pagnussat AS. Transcranial magnetic stimulation combined with upper-limb training for improving function after stroke: A systematic review and meta-analysis. *Journal of the neurological sciences*. Oct 15 2016;369:149-158. doi:10.1016/j.jns.2016.08.016

16. Hao Z, Wang D, Zeng Y, Liu M. Repetitive transcranial magnetic stimulation for improving function after stroke. *The Cochrane database of systematic reviews*. May 31 2013;(5):Cd008862. doi:10.1002/14651858.CD008862.pub2

17. Hsu WY, Cheng CH, Liao KK, Lee IH, Lin YY. Effects of repetitive transcranial magnetic stimulation on motor functions in patients with stroke: a meta-analysis. *Stroke*. Jul 2012;43(7):1849-57. doi:10.1161/strokeaha.111.649756

18. Le Q, Qu Y, Tao Y, Zhu S. Effects of repetitive transcranial magnetic stimulation on hand function recovery and excitability of the motor cortex after stroke: a meta-analysis. *American journal of physical medicine & rehabilitation*. May 2014;93(5):422-30. doi:10.1097/phm.0000000000000027

19. Ille S, Kelm A, Schroeder A, et al. Navigated repetitive transcranial magnetic stimulation improves the outcome of postsurgical paresis in glioma patients - A randomized, double-blinded trial. *Brain Stimul*. Jul-Aug 2021;14(4):780-787. doi:10.1016/j.brs.2021.04.026

20. Kirton A, Andersen J, Herrero M, et al. Brain stimulation and constraint for perinatal stroke hemiparesis: The PLASTIC CHAMPS Trial. *Neurology*. May 3 2016;86(18):1659-67. doi:10.1212/WNL.0000000000002646

21. Krieg SM, Lioumis P, Makela JP, et al. Protocol for motor and language mapping by navigated TMS in patients and healthy volunteers; workshop report. *Acta Neurochir (Wien)*. Jul 2017;159(7):1187-1195. doi:10.1007/s00701-017-3187-z

22. Spetzler RF, Martin NA. A proposed grading system for arteriovenous malformations. *J Neurosurg*. Oct 1986;65(4):476-83. doi:10.3171/jns.1986.65.4.0476

23. Gomez L, Vidal B, Morales L, et al. Low frequency repetitive transcranial magnetic stimulation in children with attention deficit/hyperactivity disorder. Preliminary results. *Brain Stimul*. Sep-Oct 2014;7(5):760-2. doi:10.1016/j.brs.2014.06.001

24. He Y, Zhang Q, Ma TT, et al. Effect of repetitive transcranial magnetic stimulation-assisted training on lower limb motor function in children with hemiplegic cerebral palsy. *BMC Pediatr*. Feb 22 2024;24(1):136. doi:10.1186/s12887-024-04605-5

25. Marzbani H, Shahrokhi A, Irani A, Mehdinezhad M, Kohanpour M, Mirbagheri MM. The Effects of Low Frequency Repetitive Transcranial Magnetic Stimulation on White Matter Structural Connectivity in Children with Cerebral Palsy. *Annu Int Conf IEEE Eng Med Biol Soc*. Jul 2018;2018:2491-2494. doi:10.1109/EMBC.2018.8512866

26. Rossi S, Hallett M, Rossini PM, Pascual-Leone A, Safety of TMSCG. Safety, ethical considerations, and application guidelines for the use of transcranial magnetic stimulation in clinical practice and research. *Clin Neurophysiol*. Dec 2009;120(12):2008-2039. doi:10.1016/j.clinph.2009.08.016

27. Rossi S, Antal A, Bestmann S, et al. Safety and recommendations for TMS use in healthy subjects and patient populations, with updates on training, ethical and regulatory issues: Expert Guidelines. *Clin Neurophysiol*. Jan 2021;132(1):269-306. doi:10.1016/j.clinph.2020.10.003

28. Anderson V, Spencer-Smith M, Wood A. Do children really recover better? Neurobehavioural plasticity after early brain insult. *Brain*. Aug 2011;134(Pt 8):2197-221. doi:10.1093/brain/awr103

29. Johnston MV. Plasticity in the developing brain: implications for rehabilitation. *Dev Disabil Res Rev*. 2009;15(2):94-101. doi:10.1002/ddrr.64

30. deVeber GA, MacGregor D, Curtis R, Mayank S. Neurologic outcome in survivors of childhood arterial ischemic stroke and sinovenous thrombosis. *J Child Neurol*. May 2000;15(5):316-24. doi:10.1177/088307380001500508

31. Kim CT, Han J, Kim H. Pediatric stroke recovery: a descriptive analysis. *Arch Phys Med Rehabil*. Apr 2009;90(4):657-62. doi:10.1016/j.apmr.2008.10.01
